# Perspectives and Knowledge about Fertility Preservation Strategies among Female Cancer Patients in Turkey

**DOI:** 10.1155/2023/6193187

**Published:** 2023-02-08

**Authors:** Volkan Emirdar, Volkan Karataşli, Ferruh Acet, Gulin Okay, Funda Gode, Alaattin Karabulut, Çağatay Arslan

**Affiliations:** ^1^Department of Obstetrics & Gynecology, Izmir Economy University, School of Medicine, Medical Park Hospital, Izmir, Turkey; ^2^In Vitro Fertilization Unit, Izmir Economy University, Medical Park Hospital, Izmir, Turkey; ^3^Department of Obstetrics & Gynecology, University of Health Science Tepecik Education and Research Hospital, Izmir, Turkey; ^4^Department of Obstetrics & Gynecology, Ege University, School of Medicine, Izmir, Turkey; ^5^Department of Medical Oncology, Izmir Economy University, School of Medicine, Medical Park Hospital, Izmir, Turkey

## Abstract

**Objectives:**

To evaluate the knowledge level and perspectives of female cancer patients regarding fertility preservation techniques before gonadotoxic treatment. *Material and Methods*. This was a prospective observational survey-based study conducted between 2016 and 2020 in Izmir Economy University Medical Park Hospital. A total of 150 female cancer patients aged 18–42 years were included. The participants completed a 17-item questionnaire, developed by the research team to evaluate their knowledge and perspectives on fertility preservation techniques.

**Results:**

The mean age of the patients was 39.5 ± 4.9 years. Only 64.7% of the patients were referred to fertility counseling by a gynecologist, while 72.6% of the patients knew of the risk of infertility after cancer treatment. There was a significant correlation between the health status and cancer stage of the patient (*p*=0.003). The estimated future chance of becoming pregnant spontaneously or through fertility preservation techniques was significantly higher in patients with a higher education level (*p*=0.041 or 0.008, respectively). Satisfaction with the counseling process was reported as high or low by 66.7% or 20% of the patients, respectively.

**Conclusions:**

The rate of referral of reproductive-age cancer patients to fertility preservation counseling is still not satisfactory. Education level was the only variable significantly associated with a motivation to become pregnant after cancer treatment, either spontaneously or through fertility preservation techniques.

## 1. Introduction

More than one million females are diagnosed with cancer annually, of whom 10% are of reproductive age [1, 2]. The gonadotoxic effects of chemotherapy and radiotherapy may lead to premature ovarian failure and infertility. Increased survival rates with newer oncologic treatments have resulted in improved quality of life and an increase in the search for fertility options after cancer treatment. Recent developments in assisted reproductive medicine have made it possible to preserve fertility after gonadotoxic cancer treatment. Despite the emphasis on fertility preservation counseling, studies have reported that only a few females of reproductive age receive counseling by a specialist, and that the timing of the counseling is often inconvenient for the patients [3–6].

Lack of adequate time before initiating treatment of the primary disease and the risk of mortality are the major factors causing a delay in fertility preservation [7]. Although specific ovarian stimulation protocols have been well-defined to avoid high levels of hormones in fertility preservation treatments of hormone receptor positive breast cancer patients, there are still remaining concerns about the recurrence or adverse effect on the tumor cells for this patients. This also may be another reason not to choose fertility preservation treatments for patients with hormone dependent tumors.

Furthermore, although clinical practice guidelines specify the fertility preservation techniques, time requirements, and efficacies, the knowledge of health care providers in the field is still limited [8]. Although multiple reasons contribute to patient perceptions, these may vary among populations. Geographic, cultural, and economic differences should be considered during fertility preservation counseling.

Several retrospective studies have investigated the efficacy of fertility preservation counseling and patient perspectives regarding this approach [9–11]. However, there are limited data about the knowledge and perspectives of cancer patients on fertility preservation techniques in our country.

### 1.1. Objectives

The aim of this study was to evaluate the knowledge levels and perspectives of female cancer patients regarding fertility preservation techniques before gonadotoxic treatment.

## 2. Material and Methods

This was an observational survey-based study of 150 newly diagnosed female cancer patients aged 18–42 years who presented to the Izmir Economy University Medical Park Hospital between 2016 and 2020. Patients were informed about the study prior to the cancer treatment, and face-to-face interviews were held with the participants. Patients who refused to participate or answer the questions were excluded. The referred patients were asked whether they had been referred to fertility preservation counseling; counseling was provided to the nonreferred patients. The satisfaction level related to the fertility preservation counseling was investigated.

The questionnaire was designed by a team of reproductive medicine, public health, and behavioral science experts of Izmir Economy University (Supplementary File 1). The questionnaire was evaluated by survey experts and physicians for relevance and comprehensibility. Auxiliary staff reviewed the survey for clarity. The questionnaire was administered to 10 patients before the start of the study. Their results were used to ensure that the questionnaire was appropriate in terms of intelligibility and effectiveness. The questionnaire consisted of 17 questions. The first eight questions were related to patient and disease characteristics (age, education level, and marital, current health, and psychological statuses), while the remaining nine questions focused on fertility preservation knowledge and perceptions (infertility risk due to treatment, chance of pregnancy after treatment either spontaneously or via fertility preservation techniques, and satisfaction with the counseling procedure). The second part of the questionnaire used a rating scale to allow semi-quantitative analysis and comparison between groups. The participants were categorized into three groups according to the rating scale scores: low/bad (score, 0–3), moderate (4–6), and high/good (7–10). The results were analyzed to assess the knowledge levels and perspectives of the patients on fertility preservation.

The study was approved by the institutional ethics committee (approval No. 2016/195). Informed consent was obtained from all the participants. This study was performed in accordance with the Declaration of Helsinki. Descriptive statistics were calculated for the demographic and socioeconomic characteristics, current health and psychological statuses, perspectives on future pregnancies, and fertility preservation treatment. The associations between ordinal variables were analyzed using Somers' delta. The remaining associations were analyzed using the chi-square test. The alpha level was set at 0.05, and statistical analysis was performed using SPSS Statistics v. 22.0 (IBM Corp., Armonk, NY, USA).

## 3. Results

A total of 150 female cancer patients were included in the study. The mean age of the patients was 39.5 ± 4.9 years. A majority of the patients were married (76.1%), had children (81.3%), had a university degree (46%), had been diagnosed with breast cancer (76.7%), and required only chemotherapy (75%). Other cancer types were lymphoma %4.7, leukemia %1.3, rectum cancer %1, and others %16.3 (skin cancer, renal cancer, thyroid cancer, osteosarcoma, and vulvar cancer).

Only 72.7% of the patients knew about the risk of infertility due to cancer treatment, and only 64.7% were referred to fertility counseling by a gynecologist (Table 1).

**Figure 1:**
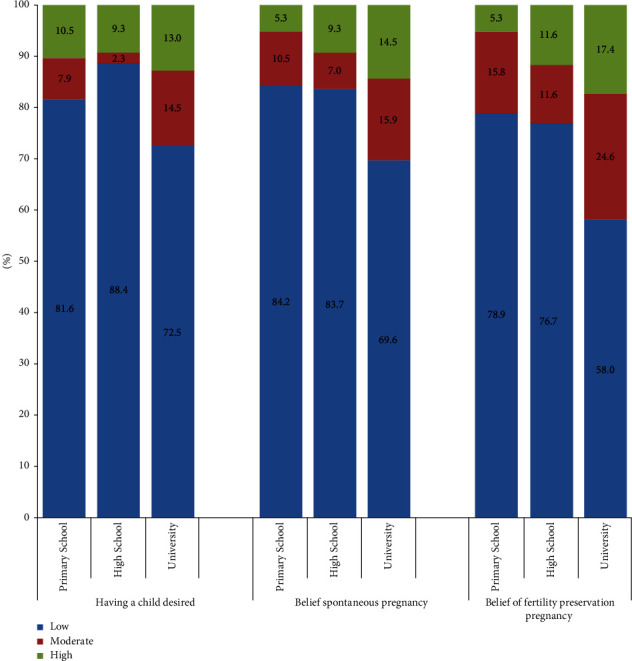
Associations of current desire to have children, chance of a future spontaneous pregnancy, and chance of a future pregnancy with fertility preservation treatment according to education level.

The overall physical and psychological health, current desire to have children, chance of a future spontaneous pregnancy, and chance of a future pregnancy using fertility preservation treatment were evaluated (Table 2). A total of 79.3% of participants believed that they had a low chance of becoming pregnant spontaneously in the future, and this belief was positively correlated with the education level (*p*=0.028) (Table 3). Answers of the higher educated patients were that they will have a higher chance of having baby in the future spontaneously or with fertility preservation methods due to their higher awareness levels. Because incomes of the patients were not questioned, therefore, this finding was associated with a higher awareness of higher educated patients, not higher income or greater economic possibilities. On the same subject, the belief of patients about having children spontaneously or via fertility preservation methods were questioned, and we asked them to rate their belief in this situation. The expected chance of pregnancy via fertility preservation treatment was low in 68.7% of the participants, and this chance was positively correlated with the education level (*p*=0.006) (Table 3). The desire to have children was not correlated with either a future spontaneous pregnancy or pregnancy via fertility preservation treatment (Figure 1). Satisfaction with the counseling process was high in 66.7% of the patients, while 20% reported being unsatisfied.

## 4. Discussion

Despite advances in fertility preservation techniques, the change in patient perspectives on life after cancer, and clinical guideline recommendations, the number of patients referred to fertility preservation has still not reached the desired level [10, 11]. Contrary to previous reports, we found that the attitude toward fertility preservation was not significantly correlated with the cancer type, cancer stage, or having previous children [12, 13]. Education level was the only significant determinant among our patients. In addition, the patient referral is low in our country, as in the rest of the world. To our knowledge, this was the first study performed in Turkey to investigate the social perspectives on this subject.

Based on the current guidelines, consideration of the infertility risk due to cancer treatments and referring the patient to a fertility specialist before cancer treatment are major steps [14, 15]. It is important to follow this process early to allow enough time for fertility preservation procedures. A study of oncologists reported that only 61% discussed the risk of infertility caused by oncologic treatments, while 45% did not routinely refer the patients to a reproductive medicine specialist [16]. Although the referral rate has increased, it still has not reached the required level [17–20]. We found that only two-thirds of the patients were referred to counseling by a gynecologist. This could be because oncologists and primary physicians do not emphasize this issue, while the patients avoid it due to anxiety or socioeconomic reasons [21].

Previous studies have investigated health status, desire to have children, knowledge about the infertility risk of cancer treatments, cancer type, and cancer stage to determine which of these factors contribute to the decision-making process [22]. In our study, none of these factors had an association with fertility preservation. This suggests that patient perspectives may depend on factors other than health parameters. Furthermore, three-quarters of the participants reported knowing of the infertility risk due to cancer treatment, and patients with higher education levels were more likely to know that getting pregnant spontaneously or via fertility preservation options is possible after cancer treatment. These findings agree with the literature [14, 23, 24]. It is believed that providing the relevant information may improve patient perspectives, planning of life after cancer, and interest in fertility preservation.

Several studies have previously evaluated the satisfaction level with and effectiveness of fertility preservation counseling. Hill et al. reported 64% satisfaction in their study of breast cancer patients, while Hill et al. demonstrated the impact of fertility preservation counseling on the decision-making process to pursue assisted reproductive techniques in breast cancer patients [25, 26]. In our study, a majority of the participants were extremely satisfied with the counseling process, but the percentage was lower than those reported in the literature. This may be explained by the feelings of anxiety and denial caused by a cancer diagnosis and an inability to process the questions and information. Application of a widespread referral and counseling system may lead to the adoption of fertility preservation treatment as part of routine cancer care.

In this study, none of the variables of having previous children, cancer type and stage, and the fear of not being able to conceive had any association with the fertility preservation treatment. This was not in agreement with the literature, and this disparity may be attributable to the social structure, lifestyle, perspectives on life, and socioeconomic factors [27].

The limitations of this study included the small number of patients limited to the west coast of Turkey, where the proportion of educated people is higher. Therefore, the results may not be representative of the entire country. There was also a lack of information regarding the fertility preservation treatments already performed as this was not the aim of the study. To our knowledge, this was the first prospective survey-based study on knowledge regarding fertility preservation and perspectives among female cancer patients in our country. In the future, it will also be necessary to evaluate the awareness and practices of physicians regarding fertility preservation practices.

## 5. Conclusion

Referral of reproductive age cancer patients was the main determinant of receiving fertility preservation counseling. There was no prognostic association of the factors evaluated, including cancer type, cancer stage, having previous children, and marital status, with fertility preservation treatments. It is crucial to increase the awareness of fertility preservation techniques among health care providers.

## Figures and Tables

**Table 1:** Patient characteristics.

	*n* = 150	%
Age (years), mean (±SD)	39.5 (±4.9)	

*Marital status*		
Single	19	12.7
Married	115	76.7
Widowed	16	10.6

*Previous children*		
No	28	18.7
Yes	122	81.3

*Education level*		
Primary school	38	25.3
High school	43	28.7
University	69	46

*Type of cancer*		
Breast cancer	115	76.7
Other	35	23.3

*Cancer stage*		
1 and 2	89	59.3
3 and 4	61	40.7

*Expected oncological treatment*		
Chemotherapy	75	50
Radiotherapy	5	3.3
Chemoradiotherapy	52	34.7
Other	18	12

*Knowledge of infertility risk after cancer treatment*		
Yes	109	72.6
No	41	27.4

*Referral to a fertility specialist before cancer treatment*		
Yes	97	64.7
No	53	35.3

SD = standard deviation.

**Table 2:** The current health and psychological statuses, future chance of pregnancy with or without fertility preservation treatment, and satisfaction with counseling among the participants.

	*N* = 150	%
*Current health status*		
Bad	4	2.7
Moderate	39	26
Good	107	71.3

*Current psychological status*		
Bad	15	10
Moderate	50	33.3
Good	85	56.7

*Current desire to have children*		
Low	119	79.3
Moderate	14	9.3
High	17	11.3

*Future chance of spontaneous pregnancy*		
Low	116	77.3
Moderate	18	12
High	16	10.7

*Future chance of pregnancy with fertility preservation treatment*		
Low	103	68.7
Moderate	28	18.7
High	19	12.7

*Satisfaction with fertility preservation counseling*		
Low	30	20
Moderate	20	13.3
High	100	66.7

**Table 3:** Cancer stage, type, and education level according to the chance of a future pregnancy via spontaneously or fertility preservation treatment.

	Spontaneous pregnancy chance (%)				Pregnancy chance with fertility preservation treatment (%)			
	Low	Moderate	High	p	Low	Moderate	High	p
	%	%	%		%	%	%	
*Education level*								
Primary school	84.2	10.5	5.3	**0.041**	78.9	15.7	5.4	**0.008**
High school	83.7	7.0	9.3		76.7	11.6	11.6	
University	69.6	15.9	14.5		58.0	24.6	17.4	

*Type of cancer*								
Breast cancer	75.7	13.9	10.4	0.426	67.8	18.3	13.9	0.705
Other	82.8	5.8	11.4		71.4	20	8.6	

*Cancer stage*								
1	80.5	4.9	14.6	0.707	70.7	14.6	14.6	0.428
2	70.8	16.7	12.5		58.3	27.1	14.6	
3	84.4	15.6	0.0		78.1	15.6	6.3	
4	77.8	11.1	11.1		74.1	11.1	14.8	

Bold values=*p* < 0.05.

## Data Availability

The data that support the findings of this study are available upon request from the corresponding author upon reasonable request.

## Supplementary Materials

Supplementary Materials Supplementary file 1: 17 item-questionnaire.
