# Supplementary material for: Perspectives and Knowledge about Fertility Preservation Strategies among Female Cancer Patients in Turkey
Source: Int J Clin Pract. 2023 Feb 8;2023:6193187. doi: 10.1155/2023/6193187 (PMC9931481; doi:10.1155/2023/6193187)
Supplement: Supplementary Materials — Supplementary file 1: 17 item-questionnaire. [file 6193187.f1.docx]

**QUESTIONNAIRE**

PATIENT ID:

1) AGE:

2) MARITAL STATUS:

- SINGLE MARRIED WIDOWED

3) HAVING CHILDREN BEFORE :

NO YES 🡪 HOW MANY CHILDREN YOU HAVE ? ………

4) EDUCATION LEVEL:

PRIMARY SCHOOL HIGH SCHOOL UNIVERSITY

5) CANCER TYPE:

BREAST CANCER LYMPHOMA LEUKEMIA OTHERS

(……..)

6) STAGE OF CANCER:

STAGE 1 STAGE 2 STAGE 3 STAGE 4

7) EXPECTED ONCOLOGICAL TREATMENT :

- CHEMOTHERAPY
- RADIOTHERAPY

- CHEMOTHERAPY + RADIOTHERAPY
- OTHERS
- NO NEED FOR ADDITIONAL TREATMENT

8) DO YOU KNOW THAT PLANNED CHEMOTHERAPY AND/OR OTHER TREATMENTS MAY CAUSE INABILITY TO HAVE CHILDREN IN THE FUTURE ?

YES NO

9) HAVE YOU BEEN REFERRED TO A GYNECOLOGIST FOR POSSIBLE INFERTILITY RISKS IN THE FUTURE DUE TO CANCER TREATMENTS ?

YES NO

10) WHO DIRECTED YOU FOR FERTILITY PRESERVATION CONSULTANCY ?

ONCOLOGIST FRIENDS INTERNET OTHERS

(…………..…………..)

11) HOW DO YOU RATE YOUR CURRENT HEALTH STATUS ? (1-10)

(1 = very bad, 10 = very good )


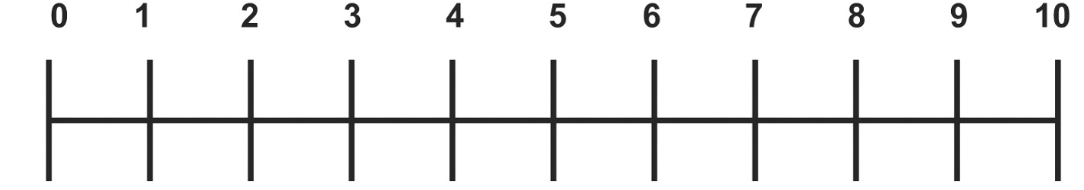


12) HOW DO YOU RATE YOUR CURRENT PSYCHOLOGICAL STATUS ? (1-10)

(1 = very bad, 10 = very good )


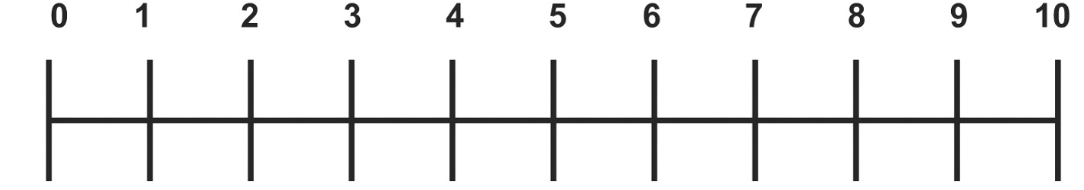


13) HOW DO YOU RATE YOUR CURRENT DESIRE TO HAVE CHILDREN?

(1-10) (1 = very bad, 10 = very good )


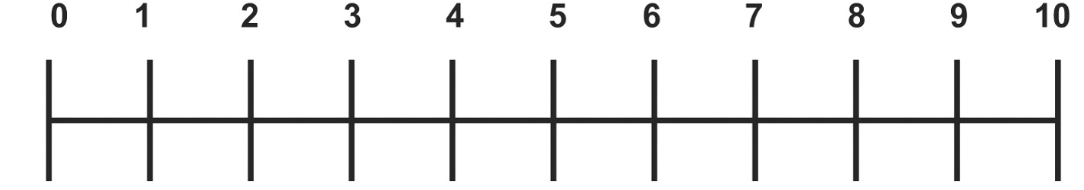


14) HOW DO YOU RATE YOUR FUTURE CHANCE OF HAVING CHILDREN SPONTANEOUSLY ? (1-10) (1 = very bad, 10 = very good )


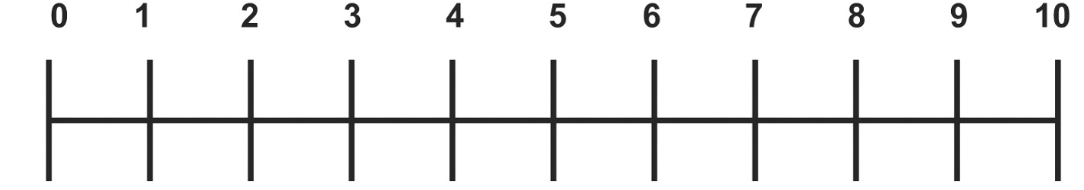


15) HOW DO YOU RATE YOUR FUTURE CHANCE OF HAVING CHILDREN WITH FERTILITY PRESERVATION TREATMENTS ? (1-10) (1 = very bad, 10 = very good )


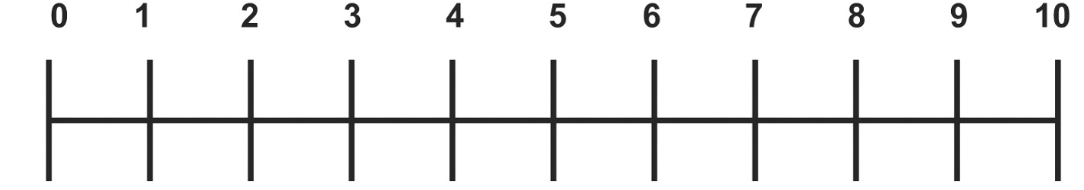


16) HOW DO YOU RATE YOUR SATISFACTION ABOUT FERTILITY PRESERVATION COUNSELING ? (1-10) (1 = very bad, 10 = very good )


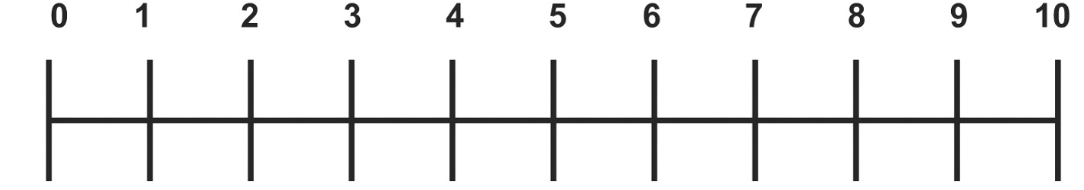


17) THE REASON FOR NOT TO CHOOSE A FERTILITY PRESERVATION OPTION ?

………………………….
